# New Insights into the Phylogeographic History of *Dirofilaria immitis* in the Canary Islands, Spain

**DOI:** 10.3390/ani15121694

**Published:** 2025-06-08

**Authors:** Rodrigo Morchón, Alfonso Balmori-de la Puente, Manuel Collado-Cuadrado, Iván Rodríguez-Escolar, Noelia Costa-Rodríguez, Elena Infante González-Mohino, Elena Carretón, José Alberto Montoya-Alonso

**Affiliations:** 1 Internal Medicine, Faculty of Veterinary Medicine, Research Institute of Biomedical and Health Sciences (IUIBS), University of Las Palmas de Gran Canaria, 35413 Las Palmas de Gran Canaria, Spain; rmorgar@usal.es (R.M.); noelia.costa@ulpgc.es (N.C.-R.); elena.carreton@ulpgc.es (E.C.); alberto.montoya@ulpgc.es (J.A.M.-A.); 2 Zoonotic Diseases and One Health Group, Biomedical Research Institute of Salamanca (IBSAL), Centre for Environmental Studies and Rural Dynamization (CEADIR), Faculty of Pharmacy, University of Salamanca, 37007 Salamanca, Spain; manuelcollado@usal.es (M.C.-C.); ivanrodriguez@usal.es (I.R.-E.); elena.igm4@usal.es (E.I.G.-M.)

**Keywords:** *Dirofilaria immitis*, dogs, PCR, hyperendemic island, Gran Canaria, Spain

## Abstract

Heartworm disease (*Dirofilaria immitis*) is an important zoonotic infection of major clinical importance in dogs widespread, and transmitted by culicid vectors. Although *D. immitis* mostly affects dogs with an overall low incidence, some islands of the Atlantic archipelagos such as the island of Gran Canaria (Canary Islands, Spain) had one of the highest historical prevalence/seroprevalence values in dogs, cats and humans. Molecular tools allow us to perform species identification diagnosis, phylogeographic and population genetics analysis that can provide key information about the factors making the disease still a threat (uncover untreated range of hosts, putative origin, etc.). In this study, we have optimized primers to amplify mitochondrial (COI, 12S) and nuclear (ITS) molecular markers from adult *D. immitis* worms. The genetic diversity and structure of *D. immitis* at the global level is limited, especially when compared with results obtained for other species of the same genus, such as *D. repens*. New minor haplotypes in the mitochondrial COI marker have been identified from adult *D. immitis* worms from infected dogs from the hyperendemic island of Gran Canaria, suggesting that the disease may have originated locally or may have been introduced from the mainland in historical times and evolved in isolation. To obtain a more complete understanding of its evolutionary history, structure and genomic diversity, comparative studies using next-generation sequencing data from endemic areas are needed, which will help in the long term to implement monitoring and control measures in a given area and to better understand its global phylogeographic history.

## Simple Summary

*Dirofilaria immitis* is a blood parasite that mainly affects dogs, cats, and humans accidentally, and is transmitted by culicid vectors. In diagnosis, the use of molecular markers is key to identify the species, especially when it cannot be clearly determined by its morphological characteristics. In this study, primers to amplify mitochondrial and nuclear genetic markers have been optimized that will help to implement control measures and to better understand the phylogeographic history of the disease on the island of Gran Canaria, a hyperendemic area of dirofilariosis.

## 1. Introduction

The nematode *Dirofilaria immitis* is the causal agent of heartworm disease, one of the most important zoonotic infections with high clinical relevance and prevalence in dogs, transmitted through culicid vectors (*Culex* spp., *Aedes* spp., *Anopheles* spp.). The larvae moult into the adult form and migrate to the pulmonary arteries and right heart ventricle, causing chronic circulatory and respiratory problems, and forming ectopic nodules in a minority of cases [1,2]. In humans, it causes human pulmonary dirofilariosis by the development of benign, asymptomatic pulmonary nodules; humans are an accidental host [3]. On the other hand, *Dirofilaria repens*, while less pathogenic in dogs, is the most common agent of human dirofilariosis, characterized by other related symptomatology causing subcutaneous nodules [4,5].

Dirofilariosis caused by *D. immitis* is present worldwide and has been extensively studied in both the New World and the Old World [2,4,6,7,8,9,10,11]. In Europe, a remarkable expansion has been noticed from southern countries considered endemic (e.g., Spain, Portugal, Italy and France), towards center, northern and eastern countries as a consequence of several factors including globalization, movement of pets, climate change and the expansion of vectors to new areas [9]. In the territories of Spain and Portugal, high levels of incidence variability can be observed between regions due to bioclimatic factors, with outdoor dogs generally at more at risk for contracting the disease [11,12,13]. In fact, although *D. immitis* mostly affects dogs with an overall low incidence (~6.5% and ~5.9% in Spain and Portugal, respectively), some islands of the Atlantic archipelagos harbor the highest prevalence. Specifically, the island of Gran Canaria had one of the highest historical prevalence/seroprevalence values in dogs, cats and humans [14,15]. Although the situation has improved as a result of control and prevention measures, the stability in recent years (>15% prevalence in dogs and cats) points to undetected reservoirs still present in the area [15], which should be further monitored in both domestic and wild species using different approaches.

Detection and diagnostic techniques used for the identification of dirofilariosis include biopsies, serological and molecular analyses. The latter involve different steps including DNA extraction, amplification, sequencing and phylogenetic inference. For instance, differential-diagnostic molecular applications have been developed to identify the parasite in definitive hosts and potential vectors [16,17,18,19,20], making this tool of great importance for the control and diagnosis of the disease. However, there are still some unresolved cases of abnormal dirofilariosis infections that highlight the need for optimizing genetic markers and improving the genomic information available to understand the origin, real impact of the disease and predict future scenarios of transmission [21,22].

Previous worldwide phylogeographic studies of *D. immitis* based on the COI gene revealed very low genetic variation, with just one haplotype sequenced in worms from multiple definitive hosts and vectors [23]. Other studies performed with more mitochondrial markers from different European/Asian countries showed low genetic variability of *D. immitis* compared with its closely related species *D. repens* [24,25]. It revealed a dominant haplotype in Europe, which was exclusive in Spain, but also uncovered some structure identifying segregated haplotypes and more diversity in some countries, such as Italy [24]. Information on these population parameters is relevant to determine the isolation and historical presence of the disease in the area. Other studies performed in America chose the 12S ribosomal RNA marker for molecular identification and the nuclear internal transcribed spacer (ITS) marker to assess genomic diversity [7,25]. Collecting more *D. immitis* DNA information from these markers is still important to gain more knowledge of the global evolution of the disease and specifically of the phylogeographic history in new areas of interest [15].

Our aim was to obtain and analyze the genetic structure and diversity of *D. immitis* worms in the hyperendemic island of heartworm of Gran Canaria, by optimizing the primers to amplify mitochondrial (COI, 12S) and nuclear (ITS) molecular markers and compare the sequences with those available in databases to gain new information about the phylogeography of the species in the area.

## 2. Materials and Methods

### 2.1. Gran Canaria

Gran Canaria is one of the seven main islands of the Canary Islands, located in the Atlantic Ocean off the northwest coast of Africa, and politically belongs to Spain. The island has a surface area of approximately 1560 km^2^ and displays a rugged orography with a central mountainous massif, creating marked climatic differences between the northern and southern regions. Gran Canaria has a subtropical climate characterized by mild temperatures year-round (average annual temperature around 21 °C) and a bimodal precipitation pattern with most rainfall occurring between October and March. The northern part of the island tends to be more humid, while the south remains relatively arid due to the influence of trade winds and the orographic barrier effect. These environmental conditions, together with a large population of domestic dogs and cats (203,917 dogs and 31,758 cats) [26] and the presence of mosquito vectors (*Culex theileri* being one of the main transmitting species) [27], favor the persistence and transmission of *Dirofilaria immitis* on the island. Several studies have documented a high prevalence of canine dirofilariosis in Gran Canaria (15.81%), and a seroprevalence of 17.2% in cats, a host in which the disease is often underdiagnosed. Moreover, there is a risk of zoonotic transmission to humans, with an average reported seropositivity of 8.27% in 2020, with the highest values in the temperate mild zone climate [15].

### 2.2. Sampling and DNA Extraction

A total of twenty-one live adult *D. immitis* worms (ten males and eleven females) from eleven infected dogs were opportunistically collected in two veterinary clinics in the north of Gran Canaria island during 2024 (February–October). The animals arrived at the respective clinics for routine treatment, not for use in this study. The worms were washed in sterile PBS pH 7.2, sexed based on diagnostic tail morphology, and stored at −20 °C in individual Eppendorf tubes filled with ethanol 99%. DNA extractions were performed inside a sterile UV irradiation hood to avoid contamination. Approximately 1 cm length piece from the worm was cut and processed with the DNeasy Blood & Tissue Kit (QIAGEN, The Netherlands, Hulsterweg) following the recommended protocol applying a digestion time interval between 1.5 h and 3 h and eluting in a final volume of 50 µL to increase concentration. Extraction blanks were always present in order to detect any possible contamination at each step. Additionally, pre-PCR procedures were also developed inside the UV hood.

### 2.3. PCR Amplification and Sequencing

For genotyping *Dirofilaria immitis* worms, we amplified two mitochondrial genes and one nuclear marker. Among the mitochondrial genes, the cytochrome c oxidase subunit I—COI was divided in 2 fragments to improve the amplification success. We used the species-specific primers for *D. repens* applied in Alsarraf et al. [24,25], with some modifications to account for *D. immitis* variability in the hybridization region of the complete mitochondrial genomes published as NC_005305 and AJ537512 [28]. In addition, we genotyped the mitochondrial 12S rRNA using the conservative markers developed for filarial nematodes in Casiraghi et al. [29]. Finally, we amplified the nuclear internal transcribed spacer (ITS) using the species-specific reverse primer designed by Thanchomnang et al. [30], and a new primer optimized in this study to skip regions with indels (Table 1).

PCR reactions were performed in a final volume of 20 μL with 2 μL genomic DNA (1 µL for 12S), 0.5 U Taq DNA polymerase, 0.2 mM dNTPs and 1 μM of each primer. The cycling conditions were slightly adapted from those originally described in order to improve PCR performance [18,31,32]. For the COI gene, we included an initial denaturation step of 300 s at 95 °C, followed by 40 cycles of denaturation (45 s at 95 °C), annealing (60 s at 56 °C for COI_I, 61 °C for COI_II) and extension (60 s at 72 °C), as well as a final extension of 7 min at 72 °C. In the case of 12S gen, we applied an initial denaturation step of 60 s at 92 °C, followed by 40 cycles of denaturation (30 s at 92 °C), annealing (45 s at 57 °C) and extension (60 s at 72 °C), as well as a final extension of 10 min at 72 °C. Finally, we used the following cycling parameters for ITS: initial denaturation at 94 °C for 300 s, followed by 35 cycles of denaturation at 94 °C for 30 s, annealing at 50 °C for 30 s, extension at 72 °C for 1 min, and final extension at 72 °C for 5 min. Amplicons were visualized in an agarose gel (2%) using TBE buffer (1%), purified using Exo I/rAP and sequenced at the Sanger sequencing service of the University of Salamanca with the two primers per sample. Both sequence directions from every sample were assembled using Geneious Prime 2023.2.1 [33].

### 2.4. Phylogenetic, Genetic Diversity, Differentiation and Statistical Analyses

All available *D. immitis* sequences from each marker in GenBank repository were downloaded using the package interface ‘rentrez’ in R v4.2.3 [34]. Both COI fragments were concatenated in a single sequence for subsequent analysis. Sequences below a threshold of homologous bases were discarded (<400 bp for 12S, <1000 for COI and <100 bp for ITS). They were renamed according to its region of origin, aligned using MAFFT 7.490 [35], and gap positions removed using Gblocks v0.91b [36]. Finally, we reconstructed a maximum-likelihood phylogenetic tree from each alignment with RAxML 8.0.0 using the GTR substitution model, GAMMA model of rate heterogeneity and executing 100 rapid bootstrap inferences [37], and the haplotype genealogies were based on these trees using Haploviewer [38]. The map showing the distribution of haplotypes by region was represented using QGIS 3.16 [39].

Nucleotide genetic diversity was calculated just for the COI region of Gran Canaria *D. immitis*, non-Gran Canaria, and for all known haplotypes of *D. immitis* as a whole. We measured Tajima’s D to test for neutral evolution, nucleotide diversity per site (π), haplotype diversity (Hd), the number of variable sites (S) using pegas 1.3 [40] and ape 5.7-1 [41] packages in R. We compared these estimates with those found for its sister species *D. repens* reported in previous studies [24,25]. Genetic differentiation (F_st_) for Gran Canaria vs. non-Gran Canaria *D. immitis* samples was calculated with the R package hierfstat 0.5-11 using the WC84 method of the *genet. dist* function [42].

## 3. Results

**Figure 1:**
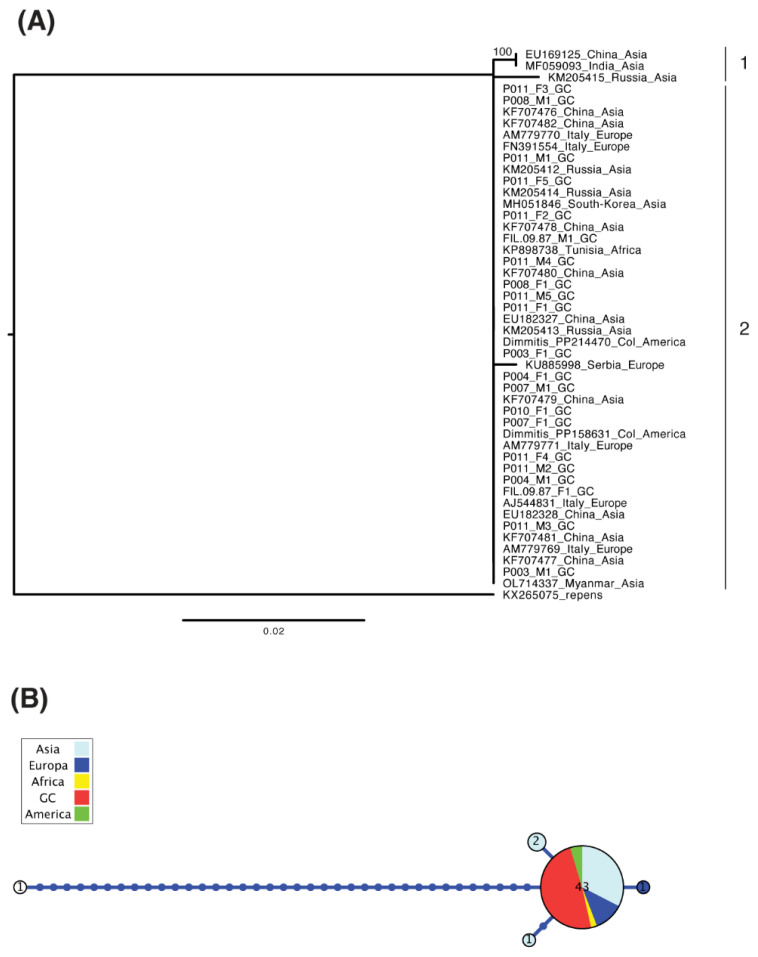
Phylogenetic information derived from the 12S gene amplified in *D. immitis* samples using *D. repens* as an outgroup, with bootstrap values shown for relevant clades mentioned in the text and scale in substitutions per position (**A**), showing the haplotype genealogy, where the size of the circles is proportional to the number of identical sequences detected, bars represent nucleotide substitutions, and the sequences derived from this study and those coming from different continents are shown with different colors (**B**). (1) Sequences from Asia showing one cluster of two sequences, (2) sequences from Europe (including sequences from Gran Canaria (GC)), Asia, America and Africa.

**Figure 2:**
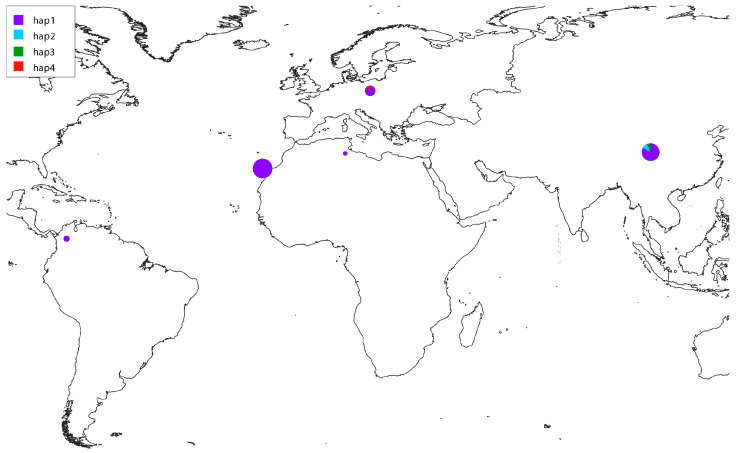
Distribution of 12S haplotypes for *D. immitis* across the different continents using a color-coded scale to indicate different haplotypes, where the size of the circles is proportional to the number of sequences available in the region.

The amplification and sequencing of twenty-one *D. immitis* samples provided 105 new sequences and 44,226 base pairs for nuclear and mitochondrial markers. The haplotypes obtained in this study have been deposited in GenBank, under the accession numbers PV469766-PV469786 for the first COI fragment (21 sequences; 671 bp), PV470952-PV470972 for the second fragment of COI (21 sequences; 652 bp), PV470166-PV470186 for 12S sequences (21 sequences; 475 bp), and PV472508-PV472574 for ITS (42 sequences—two alleles per sample—154 bp) (Supplementary Table S1). After trimming short sequences and unknown bases, alignments’ length before phylogenetic reconstruction contained 1322 bp for concatenated COI, 401 bp for 12S and 119 bp for ITS. The gene tree of the four continents with available mitochondrial 12S sequences (summing up to a total of 47 samples) from all over the world present sequences from different origins intermixed in it (Figure 1A), sharing the most frequent haplotype between them (Figure 1B). Other minor haplotypes were present in Asia and Europe. In fact, when we plot the geographic location of haplotypes in their respective continents of origin, Gran Canaria shares the haplotype with Asia, Africa, America and the rest of Europe (Figure 2).

**Figure 3:**
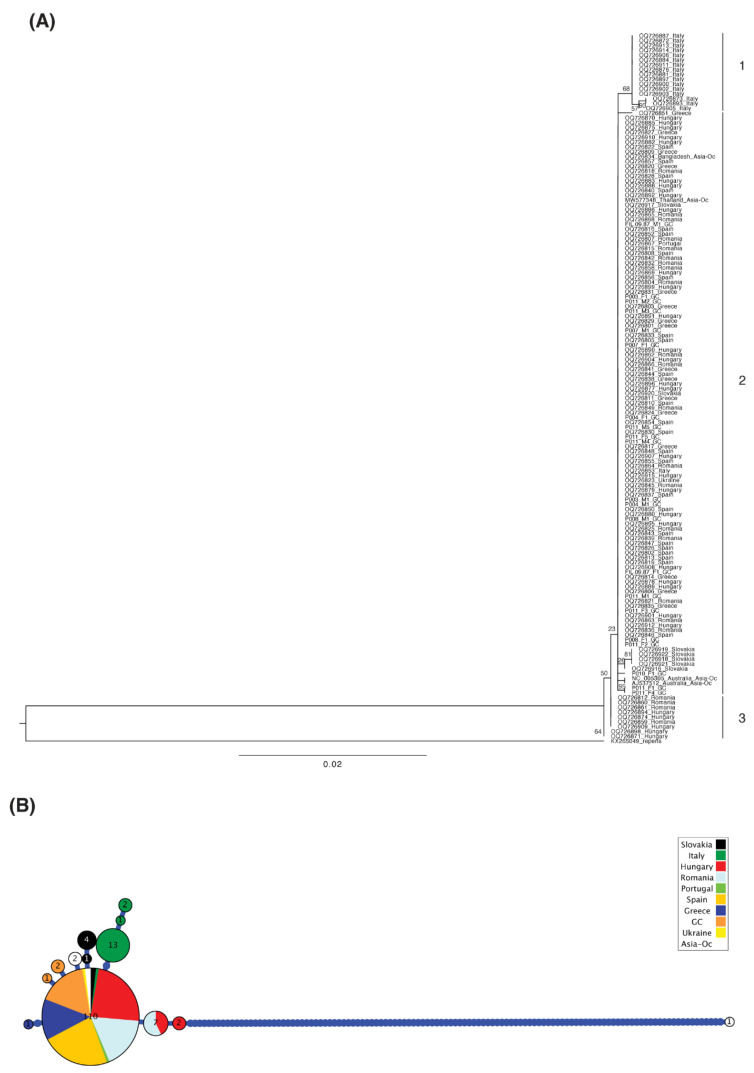
Phylogenetic information derived from COI amplified in *D. immitis* samples using *D. repens* as an outgroup, with bootstrap values shown for relevant clades mentioned in the text and scale in substitutions per position (**A**), showing the haplotype genealogy, where the size of the circles is proportional to the number of identical sequences detected, and bars represent nucleotide substitutions, and the sequences derived from this study and those coming from different countries are shown with different colors (**B**). (1) Sequences from Italy showing two nested clusters within a clade, (2) sequences from Spain (including one cluster of two sequences from Gran Canaria (GC)), Italy, Greece, Romania, Hungary, Slovakia (showing one nested cluster within a clade), Portugal, Ukraine, Asia—Oceania. (3) Sequences from Romania and Hungary showing two clusters.

**Figure 4:**
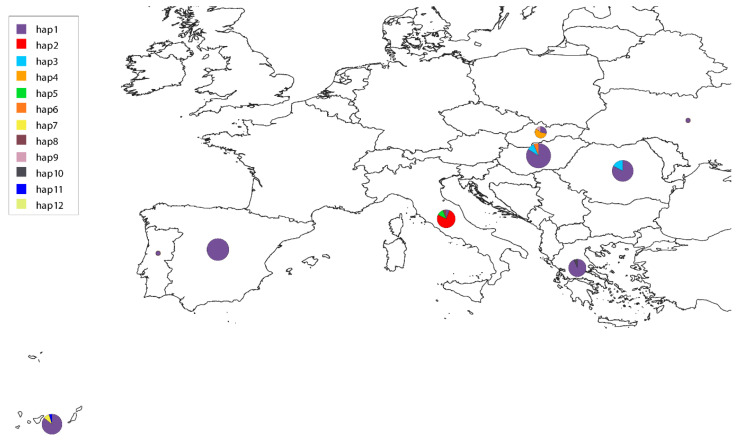
Zoom-in on the distribution of COI haplotypes for *D. immitis* across the different European countries, using a color-coded scale to indicate different haplotypes, where the size of the circles is proportional to the number of sequences available in the region. Note that the segregating haplotype from Australia (hap12) is not covered by the map.

Regarding combined COI data, we gathered 146 sequences and observed that the sequences from different regions again appeared intermixed in the gene tree, with some exceptions within Italy, Slovakia, Romania–Hungary, and Gran Canaria, which appear to form individual clades, suggesting some independent lineage evolution in those countries (Figure 3A). The Gran Canaria sequences as a whole do not form a clade, and that was also the case for the sequences available from the other regions. Gran Canaria showed three haplotypes (orange) in the first fragment, whereas only one of those haplotypes had been described in Spain until now (Figure 3B). New sequences from Gran Canaria differ in one single nucleotide polymorphism (SNP) from the major haplotype. Italy, Slovakia, Hungary, and Spain (including the Canary Islands) are the countries with the highest number of haplotypes (between three and four) (Figure 3B and Figure 4).

**Figure 5:**
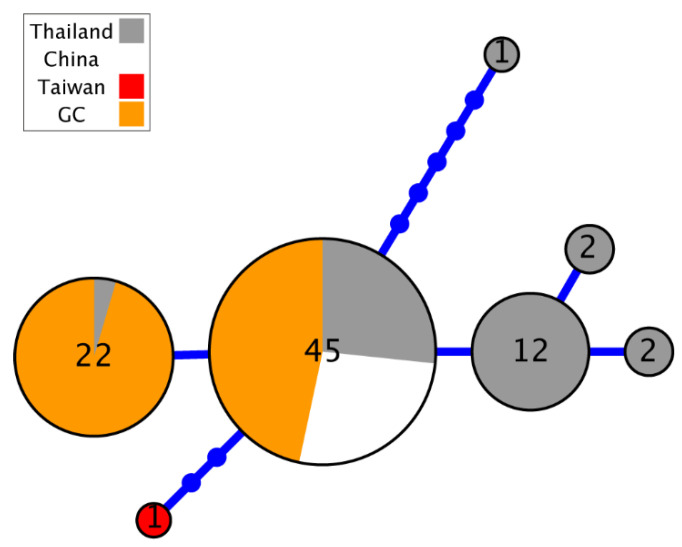
Phylogenetic information derived from ITS amplified in *D. immitis* samples showing the haplotype genealogy where the size of the circles is proportional to the number of alleles detected. All genotypes for Gran Canaria (GC) sequences were heterozygotic.

For the ITS marker, we obtained 42 new sequences (two alleles per sample) from Gran Canaria (totaling 85 with those downloaded from GenBank), with all being heterozygotic in the first base and with alleles already described in Asia (Figure 5). Unfortunately, there were no available sequences from other parts of Europe.

Neutral evolution of the COI marker was not rejected in the neutrality test, although it has an excess of rare variants (Table 2). When we measure nucleotide diversity in Gran Canaria, we observed a relevant value of π = 0.209 in comparison with the nucleotide diversity measured for the rest of the territories (π > 0.6), considering the lower geographic extension and the higher sampling effort in the region (Table 2). There is very low haplotypic diversity for *D. immitis* (Hd < 0.5) in comparison with what was found in its closely related species *D. repens* found in previous studies (Hd > 0.5). Overall, these points describe the current state of diversity in different regions, highlighting the notable diversity in Gran Canaria and other European countries compared to the general low diversity of the species elsewhere. Finally, the F_st_ distance between Gran Canaria and non-Gran Canaria was 0.045, showing low levels of genetic differentiation between populations.

## 4. Discussion

The employment of species-specific primers in biodiversity studies has multiple key applications. The identification of species, the determination of geographic or population origin, the comprehension of their phylogenetic relationships, the study of biodiversity and conservation applications are some of them [43,44]. In this study, the development and use of species-specific primers for the mitochondrial (COI, 12S) and nuclear (ITS) markers have contributed significantly to a comprehensive molecular analysis of the populations of *Dirofilaria immitis* in the hyperendemic island of Gran Canaria, where no previous studies existed. The generation of >100 new sequences and >40,000 pairs of bases represent a significant contribution to the existing global data. With this, a detailed phylogenetic and population genetic analysis has been carried out, placing the parasites in a much broader context of global parasitic diversity.

The global phylogenetic analyses revealed that the 12S haplotype most frequently found was widely distributed and shared among four continents, suggesting a unique global lineage, except for some countries. Regarding the COI marker, Italy shows a divergent cluster, as already observed in previous studies [24]. The presence of new minor haplotypes in the mitochondrial COI marker distinct in Gran Canaria, in comparison with a single major haplotype described previously for Spain [24], suggests that the disease may have experienced some drift, either because it originated there or was introduced from the continent in historical times. Gene flow for these markers between the continent and the islands seems limited, as only one out of three haplotypes was shared between regions, although this needs to be validated from the present study. Genetic diversity estimates did not coincide exactly with previous reports due to the alignment length and other uninformative markers considered [24,25]. Therefore, for the origin and evolutionary processes evaluated, more data should be collected to form the genetic structure of *D. immitis* in the Canaries.

The ITS marker, although more conserved, also revealed informative patterns. All samples analyzed were heterozygous in a diagnostic position, and their alleles coincided with those described previously in Asia [45]. Heterozygosity in the form of double peaks in the chromatogram have been detected previously in this marker, suggesting potential co-infections from different individuals and/or parasites [16]. In this study, we used individual worms directly extracted by surgical treatment, which allowed us to control this possibility. Although this marker showed limited phylogeographic resolution, consistent heterozygosity could indicate ancestral polymorphism retained or a possible mixture of related parental lineages. The ITS region, although sufficiently amplified for coding gaps and delimitation of species, may be less informative for fine-scale phylogeography unless combined with other variable nuclear genes or genomic data [46].

In the case of treating a geographically restricted region, Gran Canaria showed a nucleotide diversity relatively high for the COI marker [47,48,49,50]. This is particularly notable considering that the global diversity for *D. immitis* is generally low, apparently having passed through a bottleneck of genetic diversity or undergone low mutation rates [24,25,51]. The disparity in diversity patterns across species could reflect differences in their evolutionary histories, ranges of hosts, or ecological niches.

The geographic distribution of haplotypes reinforces the unique position of Gran Canaria in the epidemiology of dirofilariosis. Unlike the European continental regions, where the most frequently observed haplotype is coincident [24], the minor island haplotypes are distinct from the mainland ones. This could reflect a movement vectorially restricted and low rates of introduction from dogs or mosquito insects.

From a methodological point of view, this study demonstrates the utility of combining mitochondrial and nuclear markers to understand the population structure of parasites. However, the limited variability of ITS and the modest length of the 12S sequences emphasize the importance of incorporating more variable nuclear markers or genomic approaches. High-throughput sequencing could provide a greater resolution and help detect signals of selection, introgression, or historical demography to more completely understand the population dynamics of filarial parasites [22].

The findings of this study have various important implications for the epidemiology and evolutionary biology of *D. immitis*. The relatively high genetic diversity observed in Gran Canaria could indicate a cycle of ancient transmission and stability on the island, in a place of high introduction pressure. These epidemiological data precede previous findings suggesting that the island is a hyperendemic area [15]. The absence of novel or divergent haplotypes in continental regions suggests a contemporaneously limited gene flow between areas. Furthermore, the detection of widely distributed mitochondrial haplotypes at a global level, together with variants more locally adapted [52], suggests a combination of ancient dispersal events and recent regional adaptation. Understanding the zoonotic potential of *D. immitis* and its distribution in southern Europe is crucial to predict future trends and possible emergence in non-endemic regions. Similarly, the heterozygosity observed in the more conserved nuclear markers could indicate reproductive complexities or a cryptic population structure emerging in the area.

This study has some limitations, such as the number and geographic breadth of the samples. Future efforts should gather samples from other islands in the archipelago and the mainland to evaluate inter-regional gene flow. Expanding the number of adult *D. immitis* and blood samples from regions such as Africa, Europe, the Far East, North and South America would help clarify the patterns of global diversity. Other factors to consider include the use of sequencing techniques with greater resolution that reveal population structures at a fine scale, gene flow, and selection signals, the acquisition of data on the biology of vectors and the movements of definitive hosts. Obtaining information on the transmission dynamics and possible barriers to gene flow, and the monitoring of the genetic diversity of *D. immitis* in the long term, would help to detect changes related to climate, vector ecology, and control interventions.

## 5. Conclusions

In this study, mitochondrial and nuclear markers were combined to provide evidence of the notable genetic diversity of *D. immitis* in the hyperendemic area of Gran Canaria, contributing with a non-negligible haplotype and nucleotide diversity, which suggests a long evolutionary and epidemiological complexity. Although the gene flow with continental populations seems limited, the detection of both shared haplotypes and others with unique characteristics highlights the importance of insular populations in the configuration of the global genetic panorama of *D. immitis*.

New minor haplotypes have been identified in the mitochondrial COI marker, suggesting that the disease may have experienced a certain drift, either because it originated there or was introduced from the continent in historical times. However, this region does not form an independent evolutionary lineage, and the majority haplotype was shared with other countries in the mainland.

To obtain a more comprehensive understanding of its evolutionary history, structure, and genomic diversity, more comprehensive studies are needed using data that include genomic and high-throughput sequencing data, samples from different ecological and environmental requirements, and from a range of longitudes to fully understand the dynamic evolution of this parasite and orient public health strategies in endemic regions.

## Figures and Tables

**Table 1:** Mitochondrial and nuclear primers used in this study including optimizations to target *D. immitis* worms (*). The fragment length is given without counting the primers.

Name	Sequence 5′ to 3′	Fragment	Length
COI_I_F *	GGTTTATTTTTGTTATTTAGTATGAA	Mitochondrial (COX1_I)	797
COI_I_R *	TATCAGTCAAAAATAAAACACATT	Mitochondrial (COX1_I)	
COI_II_F *	GTGAATGTGTTTTATTTTTGAC	Mitochondrial (COX1_II)	755
COI_II_R *	CCCTTCTAATAACCTTCCAATGAA	Mitochondrial (COX1_II)	
12SF	GTTCCAGAATAATCGGCTA	Mitochondrial (12S)	475
12SdegR	ATTGACGGATG(AG)TTTGTACC	Mitochondrial (12S)	
ITS_F *	ACATACA(CT)ACATACAATAATA	Nuclear (ITS)	180
DI-R	GATAATCTGATCGATATTGACCCT	Nuclear (ITS)	

**Table 2:** Genetic diversity measured in the studied populations of *D. immitis* with new data provided in this study: N (number of studied sequences), H (number of haplotypes), S (number of variable sites), Hd (haplotype diversity), π (nucleotide diversity per site) and Tajima’s D test for neutrality (n.s.—non-significant, *p* > 0.5).

Population	Length	N	H	S	Hd	π × 10^3^	Tajima’s D
*D. immitis* (excluding GC)	1322	125	10	8	0.446	0.671	−0.94 (n.s.)
*D. immiitis* (GC)	1322	21	3	2	0.267	0.209	−1.16 (n.s.)
*D. immitis* (whole)	1322	146	12	10	0.423	0.614	−1.33 (n.s.)

## Data Availability

The raw data supporting the conclusions of this article will be made available by the authors without undue reservation.

## Supplementary Materials

The following supporting information can be downloaded at: https://www.mdpi.com/article/10.3390/ani15121694/s1, Table S1: List of the Genbank accession numbers generated in this study for different mitochondrial (COI, 12S) and nuclear (ITS) markers, indicating the respective length of the nucleotide sequence.
