# Supplementary material for: New Insights into the Phylogeographic History of Dirofilaria immitis in the Canary Islands, Spain
Source: Animals (Basel). 2025 Jun 8;15(12):1694. doi: 10.3390/ani15121694 (PMC12189116; doi:10.3390/ani15121694)
Supplement: Supplementary file 1 [file animals-15-01694-s001.zip › animals-3637623-supplementary.pdf]

Supplementary Table S1.

Specimen Code	Marker	Accession Number	Length
FIL.09.87_F1	COX1_I	PV469766	671
FIL.09.87_M1	COX1_I	PV469767	671
P003_F1	COX1_I	PV469768	671
P003_M1	COX1_I	PV469769	671
P004_F1	COX1_I	PV469770	671
P004_M1	COX1_I	PV469771	671
P007_F1	COX1_I	PV469772	671
P007_M1	COX1_I	PV469773	671
P008_F1	COX1_I	PV469774	671
P008_M1	COX1_I	PV469775	671
P010_F1	COX1_I	PV469776	671
P011_F1	COX1_I	PV469777	671
P011_F2	COX1_I	PV469778	671
P011_F3	COX1_I	PV469779	671
P011_F4	COX1_I	PV469780	671
P011_F5	COX1_I	PV469781	671
P011_M1	COX1_I	PV469782	671
P011_M2	COX1_I	PV469783	671
P011_M3	COX1_I	PV469784	671
P011_M4	COX1_I	PV469785	671
P011_M5	COX1_I	PV469786	671
FIL.09.87_F1	12S	PV470166	475
FIL.09.87_M1	12S	PV470167	475
P003_F1	12S	PV470168	475
P003_M1	12S	PV470169	475
P004_F1	12S	PV470170	475
P004_M1	12S	PV470171	475
P007_F1	12S	PV470172	475
P007_M1	12S	PV470173	475
P008_F1	12S	PV470174	475
P008_M1	12S	PV470175	475
P010_F1	12S	PV470176	475
P011_F1	12S	PV470177	475
P011_F2	12S	PV470178	475
P011_F3	12S	PV470179	475
P011_F4	12S	PV470180	475
P011_F5	12S	PV470181	475
P011_M1	12S	PV470182	475
P011_M2	12S	PV470183	475
P011_M3	12S	PV470184	475
P011_M4	12S	PV470185	475
P011_M5	12S	PV470186	475
FIL.09.87_F1	COX1_II	PV470952	652
FIL.09.87_M1	COX1_II	PV470953	652
P003_F1	COX1_II	PV470954	652
P003_M1	COX1_II	PV470955	652
P004_F1	COX1_II	PV470956	652
P004_M1	COX1_II	PV470957	652
P007_F1	COX1_II	PV470958	652
P007_M1	COX1_II	PV470959	652
P008_F1	COX1_II	PV470960	652
P008_M1	COX1_II	PV470961	652
P010_F1	COX1_II	PV470962	652
P011_F1	COX1_II	PV470963	652

P011_F2	COX1_II	PV470964	652
P011_F3	COX1_II	PV470965	652
P011_F4	COX1_II	PV470966	652
P011_F5	COX1_II	PV470967	652
P011_M1	COX1_II	PV470968	652
P011_M2	COX1_II	PV470969	652
P011_M3	COX1_II	PV470970	652
P011_M4	COX1_II	PV470971	652
P011_M5	COX1_II	PV470972	652
FIL.09.87_F1	ITS (allele A)	PV472508	154
FIL.09.87_M1	ITS (allele A)	PV472509	154
P003_F1	ITS (allele A)	PV472510	154
P003_M1	ITS (allele A)	PV472511	154
P004_F1	ITS (allele A)	PV472512	154
P004_M1	ITS (allele A)	PV472513	154
P007_F1	ITS (allele A)	PV472514	154
P007_M1	ITS (allele A)	PV472515	154
P008_F1	ITS (allele A)	PV472516	154
P008_M1	ITS (allele A)	PV472517	154
P010_F1	ITS (allele A)	PV472518	154
P011_F1	ITS (allele A)	PV472519	154
P011_F2	ITS (allele A)	PV472520	154
P011_F3	ITS (allele A)	PV472521	154
P011_F4	ITS (allele A)	PV472522	154
P011_F5	ITS (allele A)	PV472523	154
P011_M1	ITS (allele A)	PV472524	154
P011_M2	ITS (allele A)	PV472525	154
P011_M3	ITS (allele A)	PV472526	154
P011_M4	ITS (allele A)	PV472527	154
P011_M5	ITS (allele A)	PV472528	154
FIL.09.87_F1	ITS (allele B)	PV472554	154
FIL.09.87_M1	ITS (allele B)	PV472555	154
P003_F1	ITS (allele B)	PV472556	154
P003_M1	ITS (allele B)	PV472557	154
P004_F1	ITS (allele B)	PV472558	154
P004_M1	ITS (allele B)	PV472559	154
P007_F1	ITS (allele B)	PV472560	154
P007_M1	ITS (allele B)	PV472561	154
P008_F1	ITS (allele B)	PV472562	154
P008_M1	ITS (allele B)	PV472563	154
P010_F1	ITS (allele B)	PV472564	154
P011_F1	ITS (allele B)	PV472565	154
P011_F2	ITS (allele B)	PV472566	154
P011_F3	ITS (allele B)	PV472567	154
P011_F4	ITS (allele B)	PV472568	154
P011_F5	ITS (allele B)	PV472569	154
P011_M1	ITS (allele B)	PV472570	154
P011_M2	ITS (allele B)	PV472571	154
P011_M3	ITS (allele B)	PV472572	154
P011_M4	ITS (allele B)	PV472573	154
P011_M5	ITS (allele B)	PV472574	154
